# Current News

**Published:** 1893-08

**Authors:** 


### Current News.

---

### MEMORIAL OF THE BRITISH DENTAL ASSOCIATION TO THE GENERAL MEDICAL COUNCIL OF GREAT BRITAIN.

"WE, the undersigned, licentiates of dental surgery of the Royal College of Surgeons, desire respectfully to call the attention of the Medical Council to the following facts:

"After the passing of the Dentist's Act in 1878, and the settlement of the dental curriculum of the United Kingdom by the Medical Council, the question of the recognition of foreign diplomas for registration was raised.

"It was then on examination found that no foreign dental diploma testifies so complete and so lengthened an education as the United Kingdom qualification. But it was, we believe, considered desirable, on the grounds of professional amenity, to recognize 'for the time being' the Harvard and Michigan qualification, as those, though falling short, made the nearest approach to our own standard, thus waiving for a time equality of qualification as between United Kingdom and foreign qualifications in favor of the applicant for registration.

"The great advances which have been made from time to time in our educational standard have rendered the inequality still more marked.

"The preliminary examination demanded by these colleges is, as may be seen from their own prospectuses, of such a nature as to fall far below the standard required from the dental students of this country, and, further, these examinations are in some instances committed to the care of individuals who have no kind of responsibility nor any claim to be considered competent for such an office.

"On these grounds we beg respectfully to suggest that the Medical Council, by virtue of the power invested in it by clause 10 of the Dentist's Act, withdraw, for the time being, the right of registration which has hitherto been accorded to the American colleges.

"TO THE GENERAL MEDICAL COUNCIL."

The following signatures were appended to this memorial: John Tomes, J. S. Turner, Morton Smale, L. J. Hutchinson, W. B. Paterson, Ashley Gibbings, William Hern, Frederick Canton, Storer Bennett, Arthur S. Underwood, Joseph Walker, T. Newland Pedley, Francis Ewbank, John Ackery, J. Howard Mummery, Sidney Spokes, Charles S. Tomes.

The Educational Committee of the Medical Council, in its final report, covers very fully the entire subject. We give the following abstract:

"There is reason to believe that *certain of the American bodies have brought themselves up to the standard of Michigan and Harvard*, and that their teaching appliances are satisfactory. From personal observation the committee have been assured of the excellence of the teaching arrangements in such centres as Baltimore and Philadelphia. It appears, therefore, to the committee that injustice is done to the American bodies whose curriculum is, to say the least, equal to those of Harvard and Michigan, by the grant of special recognition to the two last-named colleges alone." (Italics ours.)

The effect of the memorial and report is made evident by the following copy of an order of the General Medical Council of Great Britain, which we referred to briefly in our last number:

"General Medical Council Office,
"299 Oxford St., London, W., May 31, 1893.

"Sir,—By order of the General Medical Council I send you herewith a copy of a report presented to the Council by its Educational Committee, wherein the Council, at its meeting on the 29th instant, passed the following resolution:

"'That the recognition of the certificates of the degrees of Doctor of Dental Medicine of the University of Harvard and Doctor of Dental Surgery of the University of Michigan by the General Council be suspended until further notice, and that the Registrar be instructed to refuse registration of such certificates.'

"I am, sir,
"Yours faithfully,
"W. J. C. Miller,
"*Registrar.*

'To Dean of the Baltimore College of Dental Surgery."

---

### PAN-AMERICAN MEDICAL CONGRESS.

*Section on Medical Pedagogics.*—The Pedagogic Section will devote its attention especially to the history of the development of medical education in America.

In the papers presented by leading teachers recent advances in methods of instruction will be considered.

The *art of teaching*, which is regarded as a study of great interest in other branches of learning, has received hitherto but little attention from the medical profession.

The Section in Medical Pedagogics will therefore be made a prominent feature of the Congress, and it is hoped that those interested in medical education will co-operate in the work of this Section by being present and by actively engaging in the discussion of subjects presented.

Any inquiries or communications may be made through the Secretaries undersigned.

J. Collins Warren, M.D., Boston, Mass.,
*Executive President.*
Charles L. Scudder, M.D., Boston, Mass.,
*English-Speaking Secretary.*
Wm. F. Hutchinson, M.D., Providence, R. I.,
*Spanish-Speaking Secretary.*

---

### AMERICAN DENTAL ASSOCIATION.

The Thirty-third Annual Session of the American Dental Association will be held in Chicago, commencing Saturday, August 12, 1893, at 10 o'clock A.M.

George H. Cushing,
*Recording Secretary.*

---

### SPECIAL NOTICE.

Chicago July 7, 1893.

For the accommodation of the different dental associations which are to meet in Chicago before the convening of the World's Columbian Dental Congress, I have secured the Kindergarten College Hall, 10 Van Buren Street, which can be used for all meetings desiring rooms.

For further information address

J. N. Crouse,
*Chairman.*

### NATIONAL BOARD OF DENTAL EXAMINERS.

The National Board of Dental Examiners will meet in a room of the building occupied by the Columbian Dental Club, No. 300 Michigan Avenue, Chicago, Friday, August 11, 1893, at 10 A.M.

The attention of delegates is called to the following, passed by the National Board, August 5, 1891.

*Resolved*, That the various State Boards of Dental Examiners be requested each year, in season for the annual meeting, to make to the Secretary a written report of their examinations, accompanied by detailed or tabulated statements.

---

### SOUTH CAROLINA STATE DENTAL ASSOCIATION.

The Twenty-third Annual Meeting of the South Carolina State Dental Association, and of the State Board of Dental Examiners, will be held at Columbia, S. C., commencing on Tuesday, August 8, 1893, at 10 A.M.

---

### NEW BY-LAWS, PAN-AMERICAN MEDICAL CONGRESS.

*Languages.*—By-Law IX. Papers may be read in any language, providing that authors of the same shall furnish the Secretary-General with an abstract, not exceeding six hundred words in length, in either of the official languages (English, Spanish, French, or Portuguese), by not later than July 10, 1893; and providing, further, that a copy of each such paper shall be furnished in either of the official languages, at or before the time of the meeting, to the Secretary of the Section before which the same shall be read. Remarks upon papers may be made in any language, providing that members making such remarks shall furnish a copy of the same in either of the official languages before the adjournment of the session.

*Publication.*—By-Law X. All papers read, either in full or by title, shall be immediately submitted for publication in the Transactions (Special Regulation 3), but authors may retain copies and publish the same at their pleasure after the adjournment of the Congress.

*Constituent Organizations.*—By-Law XI. All medical, dental, and pharmaceutical organizations, the titles of which have been transmitted with approval to the Committee on Organization, or which may hereafter be transmitted with approval to the Executive Committee, by any member of the International Executive Committee, each for his own country, shall be subject to election by the Executive Committee, approved by the President, as constituent bodies of the First Pan-American Medical Congress, and each organization thus constituted shall have the right to designate as delegates all of its members attending the Congress; but no such organization shall meet at the time and place of meeting of the Congress as a distinct body; providing, that the Secretary of each such constituent body shall furnish a list of officers and a statement of the number of members of his respective organization to the Secretary-General not later than sixty (60) days before the meeting of the Congress, and shall forward a list of delegates chosen, to reach the Secretary-General before the opening of the Congress.

By the Executive Committee.

February 22, 1893.

---

### RECENT PATENTS.

Follgwing is a list of recent patents, reported specially for the International Dental Journal.

492,056.—Method of Producing Dental Cement. Max Sichel, San Francisco, Cal. Filed May 17, 1892.

492,266.—Flexible Shaft for Dental Engines. Arthur W. Browne, Prince's Bay, N. Y., assignor to the S. S. White Dental Manufacturing Company, Philadelphia, Pa. Filed April 26, 1892.

492,432.—Dental Engine. Charles E. Rhone, Bellefonte, Pa. Filed May 11, 1892.

492,433.—Dental Engine. Charles E. Rhone, Bellefonte, Pa. Filed October 15, 1892.

492,434.—Dental Apparatus. Alvan S. Richmond, New York, N. Y., assignor to John S. Huyler, same place. Filed December 15, 1892.

492,830.—Dental Plugger. Arthur E. Peck, Minneapolis, Minn. Filed June 6, 1892.

493,289.—Attachment for Dental Engines. Adelbert H. Peck and Clarence E. Allshouse, Chicago, Ill. Filed November 27, 1891.

493,318.—Artificial Tooth. Joshua M. Twilley, Dover, Del. Filed January 12, 1893.

493,379.—Dental Chair. Aaron P. Gould, Canton, Ohio. Filed March 16, 1889.

493,431.—Electric Motor for Dental Work. Jeremiah Keller, Canton, Ohio. Filed June 6, 1892.

493,528.—Box for Tooth-Powder. Warren A. Spalding, New Haven, Conn. Filed July 18, 1892.

493,723.—Dental Appliance for Obtunding Nerves. William P. Horton, Jr., Cleveland, Ohio, assignor of one-half to Ansel B. Jones, same place. Filed August 18, 1892.

Trade-Marks.—22,441.—Dentrifice. William H. Farrand, Rochester, N. Y. Filed January 5, 1893. Essential feature, a representation of the Eiffel Tower.

22,542.—Dentifrice. The S. S. White Dental Manufacturing Company, Philadelphia, Pa., New York and Brooklyn, N. Y., Boston, Mass., Chicago, Ill., and Atlanta, Ga. Filed February 2, 1893. Essential feature, the word "Savonola."

22,544.—Tooth-Powder. The Reliance Manufacturing Company, New York, N. Y. Filed February 1, 1893. Essential feature, the word "Opaline."

---

### REDUCTION OF FARES TO INTERNATIONAL MEDICAL CONGRESS, ROME, ITALY.

Reduction in fares to members of the International Medical Congress, to be held in Rome from September 24 to October 1, 1893.

The North German Lloyd, 2 Bowling Green, New York, offers a reduction of twenty-five per cent. to members going to and coming from the Eleventh International Medical Congress, on steamer Werra, which is to sail from New York on August 5 and September 9, and on steamer Fulda, on August 19. Both these steamers sail to Genoa. The same reduction will be made for the return trips in October and November, on the same steamers, and for the Company's Saturday (off Bremen, Sunday off Southampton) steamers.

The Hamburg-American Packet Company, 37 Broadway, New York, 125 La Salle Street, Chicago, offers a reduction of twenty-five per cent., both out and return, for all its steamers during the year 1893.

The Compagnie Générale Transatlantique, 3 Bowling Green, New York, offers the rates which are allowed French officers,—that is, $63.50 for an $80 accommodation and $91.50 for a $120 accommodation.

The Provisional Committee has made arrangements with the different companies whereby special reduced prices have been granted on the railways of the countries which the members of the Congress are to traverse.

### SOUTHERN DENTAL ASSOCIATION.

The Committee of Arrangements has secured the Kindergarten College Hall, 10 Van Buren Street, for holding the business meetings of this Association in connection with the Columbian Dental Congress. The time for the meeting is fixed for Friday, August 11, at 10 A.M.

S. N. Foster,
*Secretary.*

### SPECIAL NOTICE—ASSOCIATIONS AT CHICAGO.

The meetings of the American Dental Association and the Association of Dental Examining Boards will be held in Kindergarten College Hall, No. 10 Van Buren Street, Chicago.

J. Taft.

### ILLINOIS STATE DENTAL SOCIETY.

The Twenty-ninth Annual Meeting of the Illinois State Dental Society was held, in conjunction with the Iowa State Dental Society, at Rock Island and Davenport, May 9–12, 1893. The following officers were elected for the ensuing year: Garrett Newkirk, Chicago, President; J. W. Cormany, Mt. Carroll, Vice-President; Louis Ottofy, Chicago, Secretary; W. A. Stevens, Chicago, Treasurer; F. H. McIntosh, Bloomington, Librarian.

Louis Ottofy,
*Secretary.*

### GEORGIA STATE DENTAL SOCIETY.

The Twenty-fifth Annual Meeting of the Georgia State Dental Society was held at Atlanta, Georgia, May 9, 10, 11, 12. The following officers were elected for the ensuing year: President, N. A. Williams, Valdosta; First Vice-President, W. W. Hill, Washington; Second Vice-President, C. V. Rosser, Atlanta; Treasurer, H. A. Lowrance, Athens; Recording Secretary, S. H. McKee, Americus; Corresponding Secretary, O. H. McDonald, Griffin.

*Examining Board.*—Jno. H. Coyle, Chairman, Thomasville; D. D. Atkinson, Secretary, Brunswick; A. G. Bouton, Savannah; B. H. Catchings, Atlanta; H. H. Johnson, Macon.

*Executive Committee.*—H. R. Jewett, Chairman, Atlanta; —— Barfield, Macon; —— Hopps, Savannah; —— Hanes, Cedartown; —— Simmons, Guyton.

Next place of meeting, Tybee Island, Georgia, June, 1894.

O. H. McDonald,
*Corresponding Secretary.*

---

### ENTERTAINMENT OF THE MEDICAL PROFESSION AT THE WORLD'S FAIR.

At a meeting of the Joint Committee of the Chicago Medical Profession on World's Fair Entertainment, held at the Sherman House, November, 1892, the establishment of a Bureau of Information and Service was delegated, with approval and endorsement, to Charles Truax, Greene & Co., the committee reserving to itself the duty of such social entertainment of visiting physicians during the continuance of the Exposition as may seem desirable.

This action was confirmed at the final meeting of the Joint Committee, February 25, 1893, and on application of the Practitioner's Club and the South Side Medical Club, the matter of social entertainment was delegated to them, with full authority to act in the capacity of entertaining bodies, with the retention of the chairman and its American and foreign secretaries already appointed. Chairman, Dr. Charles Warrington Earle; American Secretaries, Dr. Archibald Church, Dr. George Henry Cleveland, Dr. John C. Cook, Dr. J. C. Culbertson; British, Dr. Sanger Brown; German, Dr. F. C. Hotz; French, Dr. Fernand Henrotin; Spanish, Dr. E. J. Gardiner; Italian, Dr. A. Lagario; Swedish, Dr. K. Sandberg; Canadian, Dr. R. D. McArthur; Russian, ———.

The scope and duties of the above secretaries will be designated in the future.

C. Warrington Earle,
*Chairman.*

